# Editorial: Autonomous (re)production, learning and bio-inspired robotics workshop

**DOI:** 10.3389/frobt.2024.1513495

**Published:** 2024-11-28

**Authors:** Andy M. Tyrrell, Emma Hart, Alan Winfield, A. E. Eiben, Jon Timmis

**Affiliations:** ^1^ School of Physics, Engineering and Technology, University of York, York, United Kingdom; ^2^ Department of Computer Science, Edinburgh Napier University, Edinburgh, United Kingdom; ^3^ Bristol Robotics Lab, University of the West of England, Bristol, United Kingdom; ^4^ School of Computer Science, VU Amsterdam, Amsterdam, Netherlands; ^5^ VC's Office, Aberystwyth University, Aberystwyth, United Kingdom

**Keywords:** autonomous robot evolution, learning, bio-inspired robotics, evolution, real-time adaptation

Traditional ways of designing and building robots have involved human experience and knowledge to decide on the robot’s morphology (body) and then produce a proprietary piece of software (brain) to control the robot, usually for a specific application, or set of similar applications. Technology and progress in machine learning and bio-inspired methods for design, provide the possibility to move away from these “traditional” methods and to consider autonomous processes in the design and production of robot morphologies and brains for multiple applications, without human intervention. Through artificial evolutionary mechanisms robot populations can be autonomously created, tested in appropriate environments (simulation or on hardware, or both), and the genetic code of suitable individuals used to create the next-generation of robot morphologies and brains (hardware and software). This iterative process can continue until successful individuals have been evolved. With the latest technologies, even the fabrication process, that is, the production of new robots, can be automated in some circumstances. This opens the possibilities for robots to be evolved in unknown or inaccessible environments.

**FIGURE 1:**
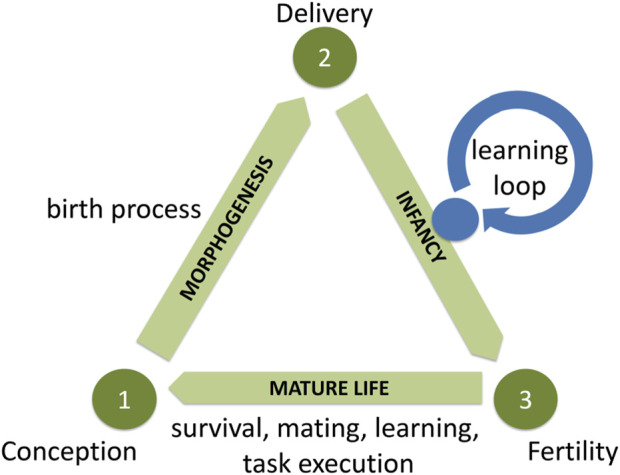
The Triangle of Life model [adapted from Eiben et al. (2013)].

A conceptual framework to provide the foundation for these ideas was first presented in Eiben et al. (2013) and illustrated in Figure 1, The Triangle of Life model. The Triangle of Life model helps illustrate these ideas and the possibilities of robots reproducing in the real world. This allows creation through different hardware approaches and different reproduction mechanisms, while focusing on the conception of a new robot organism. The other components of the Triangle capture the principal stages of such a system; the model serves as a roadmap for unlocking the potential of autonomous design and building systems where robot morphologies and controllers can evolve in real-time and real-space.

The Autonomous (re)Production, Learning and Bio-inspired Robotics workshop was held in the historic city of York 17th – 19 October 2022, supported by the EPSRC grant Autonomous Robot Evolution and inspired by The Triangle of Life. The workshop highlighted emerging trends and future directions in the field of robotics and featured invited position papers from world-leading researchers across the field, and a range of reviewed papers. Papers focused on the potential for future developments within the field of bio-inspired robotics and autonomous design and manufacture. The papers addressed areas such as: Novel methods for simultaneous evolution of morphology and control; Novel methods for facilitating learning and adaptation during lifetime; Evolution of learnability in a robot population; Investigating the balance between morphological intelligence and brain intelligence; Robot evolution in hardware; Evolution of morphologies using novel materials; Simulation of soft robots; Closing the reality gap; Evolving behavioural/morphological diversity within a robotic eco-system; Research Topic related to manufacturability and viability of robotic genotypes; Surrogate methods for fitness evaluations. This Research Topic includes three papers from that workshop and a strongly related paper to this area.

*Practical hardware for evolvable robots,* by Angus et al. This paper explores in detail the design of an example system for realising diverse evolved physical robot bodies, and specifically how this interacts with the evolutionary process. The ultimate goal of evolutionary robotics is to evolve robots that are of practical use in real-world applications. To achieve this, it is necessary to progress beyond simulation and implement in hardware, addressing the challenges that this entails. The paper examines the interplay between an evolutionary robotics process and the hardware with which the evolved robots are to be implemented. An important finding highlighted in this paper is that the evolutionary process is not separable from the hardware, since the many constraints introduced by the hardware fundamentally define the nature of the phenotype space that the evolutionary process explores.

*On Evolutionary Robotics as a modelling tool in Evolutionary Biology*, by Winfield explores the use of evolutionary robotics (ER) as a scientific instrument for addressing questions in evolutionary biology. The paper asks the question, What kind of model is an ER system?, by first using model descriptions to compare three case studies that have shed new light on the evolution of fish backbones, altruism, and modularity. The paper develops an analysis of the strengths and limitations of ER as a tool for modelling evolutionary biology followed by a review of the deeper questions in evolution and which of these might be modelled by ER. The paper concludes that that while ER is a weak model of evolution its bottom-up approach to modelling populations of evolving phenotypes and their embodied interactions does have value to biologists for testing and generating hypotheses.

*From real-time adaptation to social learning in robot ecosystems,* by Szorkovszky et al. proposes and demonstrates a novel means for social learning of gait patterns, based on sensorimotor synchronization. The paper argues that using movement patterns of other robots as inputs can drive nonlinear decentralised controllers such as Central Pattern Generators into new limit cycles, hence encouraging diversity of movement patterns. The paper demonstrates a proof of principle using a simple social learning scheme for robot gaits. It is illustrated that useful behaviours can be imitated by only communicating a series of foot contact events, such as via audible footsteps. The approach allows for multiple behaviours to be learned and switched between.

*Learning hybrid locomotion skills—Learn to exploit residual actions and modulate model-based gait control,* by Kasaei et al. proposes a locomotion framework based on a tight coupling between analytical control and deep reinforcement learning to leverage the potential of both approaches. The framework uses a model-based, full parametric closed-loop and analytical controller as a kernel to generate gait patterns. A neural network with symmetric partial data augmentation is used to automatically adjust the parameters for the gait kernel, and generate compensatory actions for all joints, augmenting the stability under unexpected perturbations. The paper indicates that the trained policies, in simulation, are robust to noise and model inaccuracies.
